# Cecal volvulus and nonrotation: two rare pathologic entities as culprits of intestinal obstruction—a case report

**DOI:** 10.1093/jscr/rjaf616

**Published:** 2025-08-12

**Authors:** Wondwosen Mengist Dereje, Menarguachew Atanaw Sisay, Gedefaye Balekew Demlie, Yohannes Leweyehu Debasu, Eyob Bayeh Tsehay, Fikadie Mengist Dereje, Asratu Getnet Amare

**Affiliations:** Department of Surgery, College of Medicine and Health Science, Gondar University, 212, Gondar 196, Ethiopia; Department of Surgery, College of Medicine and Health Science, Gondar University, 212, Gondar 196, Ethiopia; Department of Surgery, College of Medicine and Health Science, Gondar University, 212, Gondar 196, Ethiopia; Department of Surgery, College of Medicine and Health Science, Gondar University, 212, Gondar 196, Ethiopia; Department of Surgery, College of Medicine and Health Science, Gondar University, 212, Gondar 196, Ethiopia; Department of Pharmacy, Rift Valley University, 180, Addis Ababa 1245, Ethiopia; Department of Surgery, College of Medicine and Health Science, Gondar University, 212, Gondar 196, Ethiopia

**Keywords:** case report, hemi-colectomy, intestinal volvulus, laparotomy, congenital malformation, nonrotation, obstruction

## Abstract

Cecal volvulus is a rare cause of acute abdomen, accounting for 1%–3% of intestinal obstructions, and is seldom associated with intestinal nonrotation. Early diagnosis is crucial for improving outcomes, as shown in the case of a 53-year-old male with a 24-hour history of constipation, bloating, colicky pain, and bilious vomiting. He exhibited signs of severe illness, including tachycardia, fever, and abdominal distention, with blood tests showing acute inflammation. After stabilization, an emergency laparotomy revealed a necrotic, 360°-twisted cecum and abnormal intestinal positioning. The patient underwent a right colectomy with appendectomy and ileostomy, recovering uneventfully and later having the stoma reversed. Though nonrotation is usually asymptomatic in adults, it can lead to life-threatening complications like volvulus, requiring surgical intervention. This case highlights the importance of recognizing embryologic abnormalities in adults with acute abdominal symptoms to prevent severe outcomes and underscores the need for prompt surgical treatment in cecal volvulus.

## Introduction

Cecal volvulus, with an annual incidence of 2.8–7.1 cases per million individuals, accounts for only 1%–1.5% of adult intestinal obstructions [1]. Similarly uncommon is intestinal nonrotation (prevalence: 0.5%), resulting from incomplete midgut rotation during fetal development [2]. Their co-occurrence represents an extraordinary clinical scenario, with few documented instances in medical literature. This report describes a 53-year-old male presenting with cecal volvulus complicating underlying intestinal nonrotation.

## Case report

A 53-year-old male presented with a 24-hour history of obstipation progressing to abdominal distension, diffuse cramping pain, and two episodes of bilious vomiting, without prior abdominal surgery or chronic medical conditions.

Clinical examination revealed an acutely ill patient with hypotension (90/60 mmHg), tachycardia (92 bpm), and fever (38.4°C). Abdominal assessment demonstrated marked distension with hypertympanic percussion, diffuse tenderness, and an empty rectal vault. Laboratory studies revealed significant leukocytosis (22 900/mm^3^) with 95% neutrophils, while other parameters remained normal.

Following urinary catheterization and nasogastric tube placement, fluid resuscitation and intravenous antibiotics were initiated. Due to clear signs of generalized peritonitis and lack of available CT imaging, we proceeded directly to emergency laparotomy.

**Figure 1:**
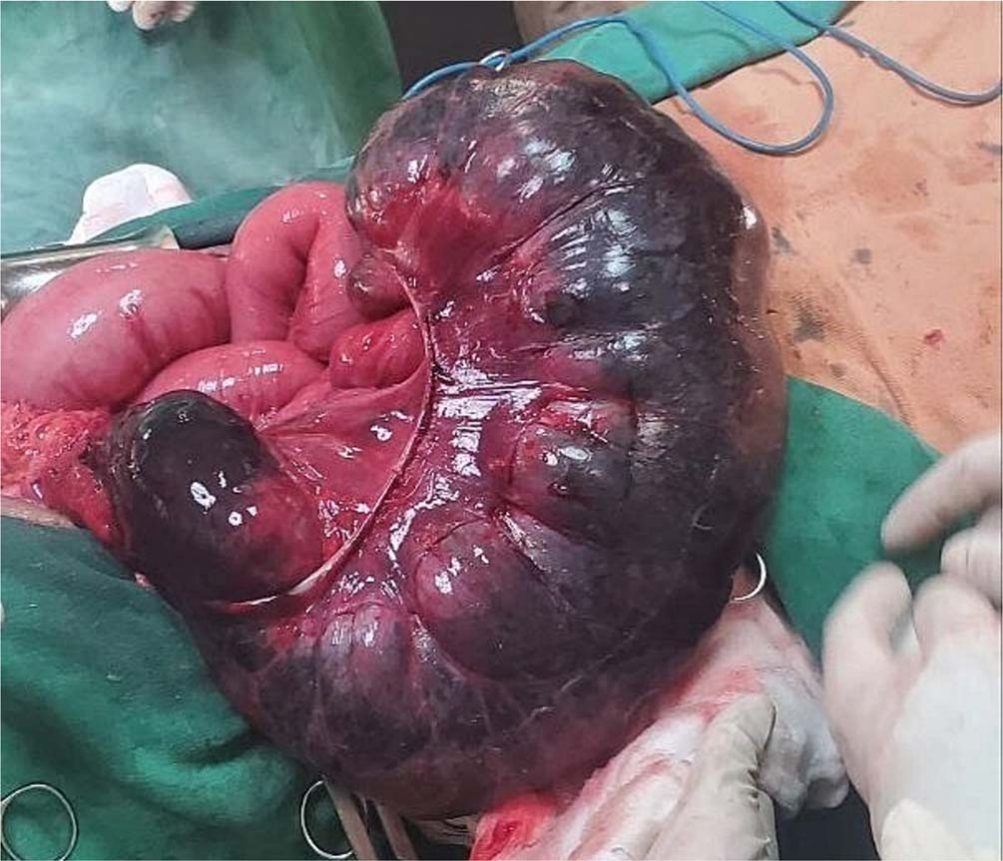
Illustration of a markedly distended cecum, demonstrating significant enlargement and clear signs of obstruction.

**Figure 2:**
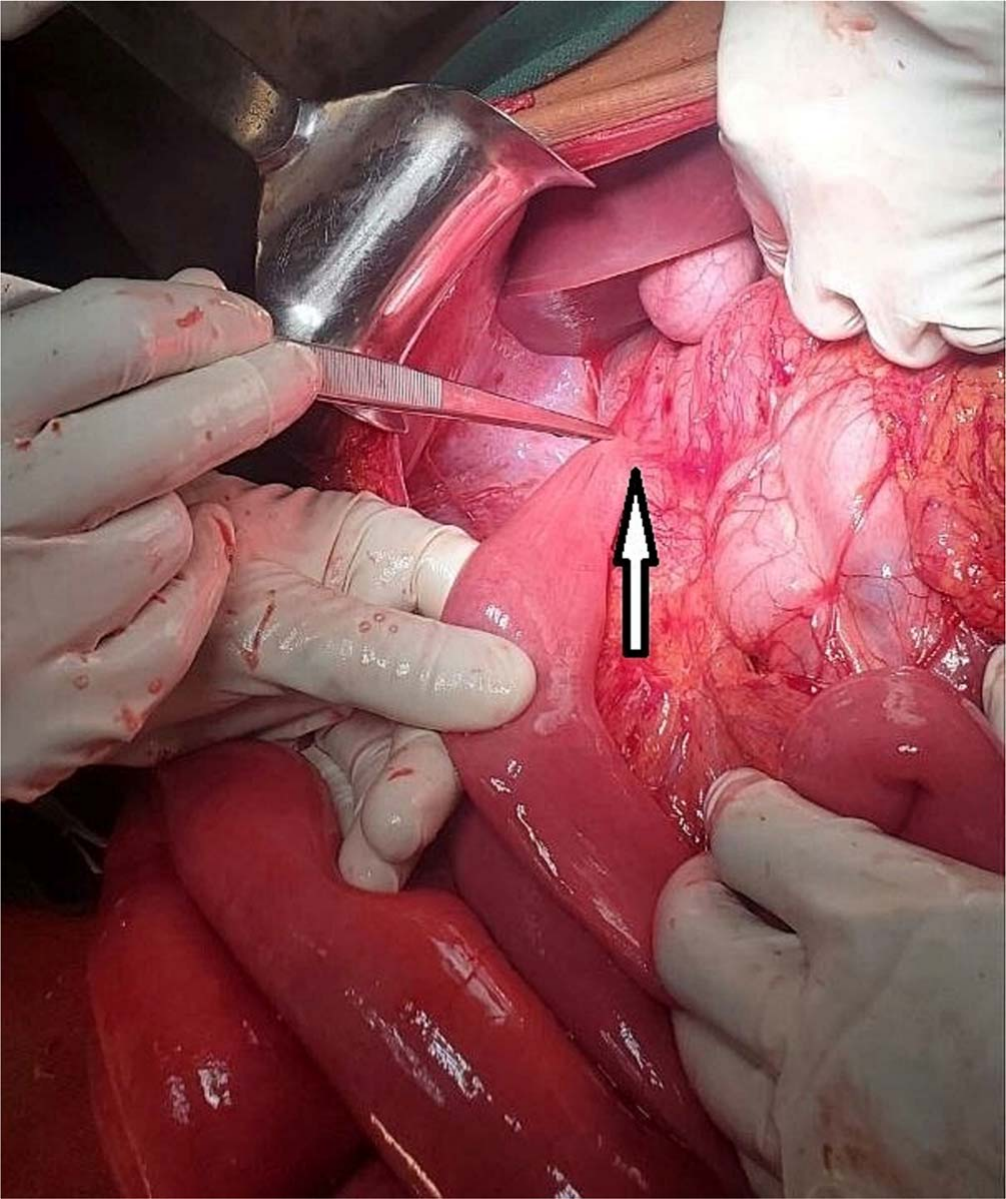
The DJJ abnormally located in the right upper quadrant. Ladd’s band is indicated by the arrow.

**Figure 3:**
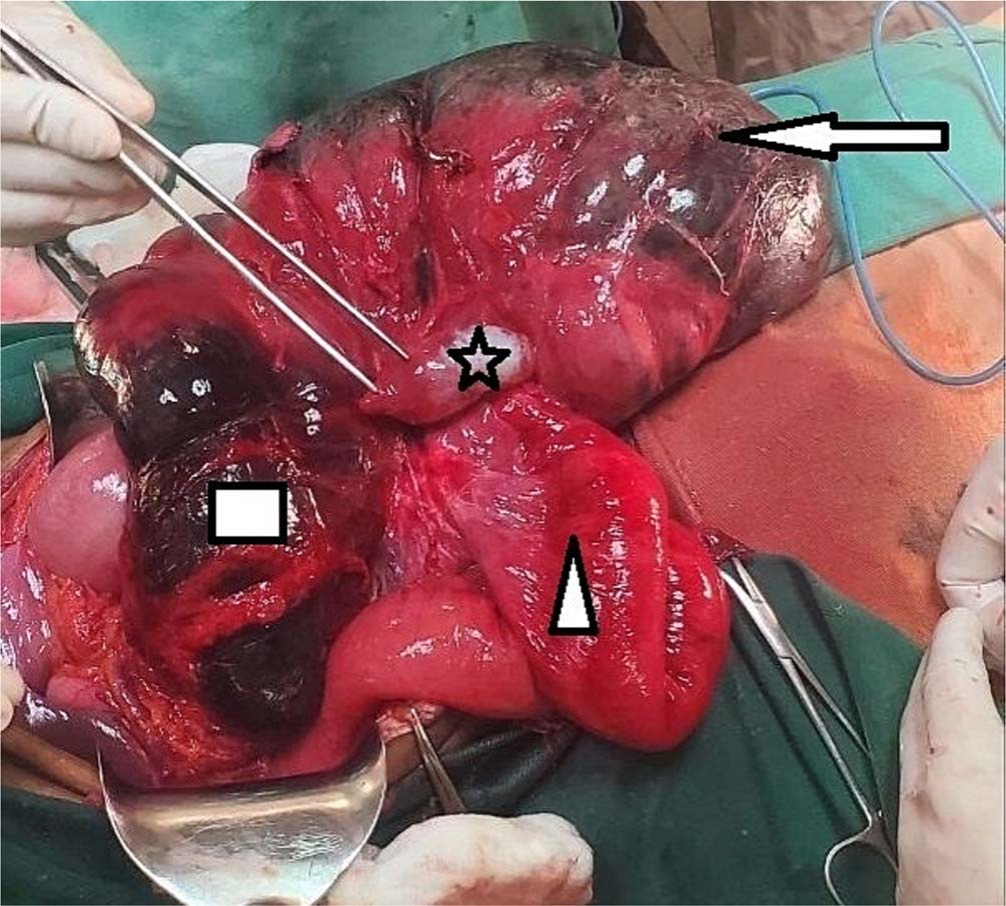
Grossly distended and detorsed cecum marked by an arrow, showing signs of severe dilation. The ileum, indicated by a triangle, appears edematous but remains viable. The appendix is identified by a star, while the ascending colon, outlined by a rectangle, shows clear signs of gangrene with necrotic tissue changes.

Surgical exploration through a midline incision identified a necrotic, 360°-twisted cecum displaced into the left hypochondrium (Fig. 1). Anatomical findings included malpositioned bowel with the duodenojejunal junction (DJJ) in the right upper quadrant (Fig. 2), small bowel confined to the right lower quadrant, and left-sided colon. Additional findings included edematous terminal ileum, gangrenous ascending colon (Fig. 3), and a Ladd’s band connecting the small bowel to the colon (Fig. 2). The patient underwent right hemicolectomy with appendectomy and end-ileostomy formation.

The patient was discharged on his fourth postoperative day with follow-up instructions. At 3 months post-surgery, he underwent ileostomy closure. He was discharged again on his fourth postoperative day after ileostomy closure with a scheduled follow-up appointment. By the second week after ileostomy closure, he remained stable and reported no complications.

## Discussion

Cecal volvulus, a rare etiology of large bowel obstruction, occurs when a mobile cecum twists axially around its mesentery or folds upward, forming a closed-loop obstruction [3]. It is the second most common location for colonic volvulus, after the sigmoid [4]. Normal midgut development is a complex process involving a 270° counter-clockwise rotation of the gut tube around the axis of the superior mesenteric artery. This rotation typically takes place between the 4th and 10th weeks of gestation, resulting in a broad-based mesentery that stretches from the ligament of Treitz in the left upper quadrant to the ileocecal valve in the right lower quadrant, with retroperitoneal fixation of the duodenum and colon [5].

When this developmental process fails, the condition is referred to as malrotation. Its prevalence is estimated at approximately 1 in 500 live births, with a higher incidence among Caucasians and a male predominance [6, 7].

Although it can be classified into several subtypes, it is often practically categorized as either nonrotation or malrotation [8]. Nonrotation occurs when the midgut returns to the peritoneal cavity without completing its normal rotation, leading to the small intestine being located on the right side of the abdomen and the colon on the left [3, 9].

Cecal volvulus is a rare condition that can cause significant morbidity if not promptly recognized and treated. It is linked to cecal hypermobility, which arises from the failure of fusion between the parietal and visceral peritoneum of the right colon. This results in a lax mesentery, allowing the cecum to fold upward (cecal bascule) or twist axially upon itself (axial cecal volvulus) [3, 7].

Patients with intestinal volvulus often have a long, redundant colonic segment and an elongated mesentery with a narrow base [10, 11]. These anatomical features can be either congenital or acquired. One congenital cause is an anomaly of intestinal rotation [12–14], which presents as a mobile (nonfixed) colon and a narrowed mesenteric stalk, often associated with Ladd’s bands or mesenteric adhesions [15].

In normal anatomy, the broad-based mesentery stabilizes the intestines and prevents volvulus. However, when the bowel is incorrectly positioned within the abdominal cavity, this mesenteric stability is lost, predisposing to volvulus, as seen in cases of nonrotation [6].

Furthermore, in adult patients with nonrotation, intermittent loose volvulus may occur, presenting with mild abdominal symptoms, such as pain and vomiting. The combination of a loose volvulus and a highly mobile mesentery can cause friction and inflammation of the mesentery. This inflammation promotes the formation of fibrous adhesions, which narrow the mesenteric stalk. These adhesions can serve as a pivot point, predisposing to acute severe volvulus at any age [15].

Nonrotation occurs in 0.2%–1% of the population, presenting several clinical challenges [5]. Adult cases make up only 0.2%–0.5% of all occurrences, leading to diagnostic difficulties and worse outcomes in adults [6].

The combination of nonrotation and cecal volvulus is exceptionally rare [9]. As a congenital anomaly, nonrotation can result in fatal colonic volvulus at any age [15]. The first description of cecal volvulus was provided by Rokitansky in 1837 [16]. While nonrotation may remain asymptomatic and be discovered incidentally, it can also result in volvulus, usually occurring at the DJJ or the mid-transverse colon [3].

When nonrotation leads to cecal volvulus, patients may present with classic symptoms of intestinal obstruction, including abdominal pain, distension, ischemia, gangrene, and perforation [17]. Over time, strangulation, ischemia, and perforation can lead to sepsis with potentially harmful consequences [3].

Diagnostic imaging, especially abdominal radiographs and CT scans, is essential for evaluating symptomatic nonrotation. Plain abdominal radiographs may show only nonspecific findings unless complications like volvulus are present [9]. When volvulus occurs, abdominal radiography typically reveals a dilated cecum and an air-fluid level.

Cecal volvulus must be considered a surgical emergency, even without clinical or radiological signs of severity. The treatment typically involves a midline laparotomy with non-oncological colectomy of the volvulized segment, most often an ileocecal resection, followed by prompt restoration of bowel continuity. Depending on the severity of ischemia in the right colon, the resection may need to encompass the entire right colon. A side-to-side stapled anastomosis is considered optimal, as it effectively addresses the luminal size difference between the ileum and the right colon [18].

Cecal gangrene requires resection, and in most cases, primary anastomosis can be performed safely. In malnourished or anemic patients, or when other factors may impair healing, an ileostomy with or without a mucus fistula may be more appropriate. Resection eliminates the risk of recurrence, and postoperative morbidity is comparable to that associated with fixation techniques [19].

Detorsion with colopexy (without resection) carries unacceptably high rates of recurrence, morbidity, and mortality, rendering it an inferior approach [20].

With recent advances in laparoscopic technology, laparoscopic colon resections are being increasingly performed. Similarly, several reports have documented the laparoscopic management of cecal volvulus [21–23].

In cases of extensive bowel necrosis or patient instability, a damage control strategy consisting of resection and stoma creation is recommended [3], as was the case in the reported patient.

In the reported case, the patient presented in a septic state. Intraoperatively, the cecum was found to be distended, and the ascending colon was gangrenous. Consequently, restoration of bowel continuity through simple ileocecal resection was deferred, and an ileostomy was performed instead. This procedure effectively relieved symptoms, and the patient experienced an uncomplicated postoperative recovery. Although both intestinal nonrotation and cecal volvulus are rare clinical conditions, prompt recognition is essential. Early surgical intervention significantly reduces the risk of serious complications such as bowel ischemia, perforation, and sepsis, all of which are associated with high morbidity and mortality.

## Conclusion

Despite being two unique pathologic entities, cecal volvulus and nonrotation can present as intestinal obstruction. A high degree of suspicion, early diagnosis, and prompt management are of paramount importance to prevent complications and save the patient.
